# Effects of Pregelatinization on the Physicochemical Properties of Corn Grits and the Quality of Cooked Waxy Corn Wrapped in Plant Leaves

**DOI:** 10.3390/foods14132287

**Published:** 2025-06-27

**Authors:** Yi Wang, Ruixuan Li, Yijiao Yan, Wanyi Niu, Yue Wang, Mingyi Shen, Ruifang Wang, Li Cheng

**Affiliations:** 1 School of Food Science and Technology, Jiangnan University, Wuxi 214122, China; 2 State Key Laboratory of Food Science and Technology, Jiangnan University, Wuxi 214122, China; 3 Collaborative Innovation Center of Food Safety and Quality Control in Jiangsu Province, Jiangnan University, Wuxi 214122, China; 4 National Engineering Research Center for Functional Food, Jiangnan University, Wuxi 214122, China

**Keywords:** pregelatinization, corn grits, cooked waxy corn wrapped in plant leaves, physicochemical properties, pasting degree, ageing resistance, starch structure

## Abstract

In this study, the effects of pregelatinization on the physicochemical properties of corn grits and the quality of cooked waxy corn wrapped in plant leaves were investigated. This investigation was conducted to address the issues of partial gelatinization and poor texture in corn grits when applied to food processing such as cooked waxy corn wrapped in plant leaves. After the corn grits were soaked at 55 °C, they were steamed for 30 min and dried at 45 °C (steam temperature maintained at 100 °C), reaching a gelatinization degree of 48.28%. The modified grits were characterized using Rapid Visco Analyzer (RVA), differential scanning calorimetry (DSC), X-ray diffraction (XRD), Fourier-transform infrared spectroscopy (FTIR), and scanning electron microscopy (SEM) to analyze pasting properties, retrogradation behavior, crystallinity, molecular structure, and morphology. The results showed that pregelatinization significantly reduced setback viscosity (from 274.83 to 154.52 mPa·s), crystallinity (from 11.12% to 3.62%), and retrogradation tendency while improving solubility, swelling power, and water-holding capacity. When used in cooked waxy corn wrapped in plant leaves, pregelatinized grits enhanced the gelatinization degree (96.11%), texture (reduced hardness by 19.49%, increased chewiness and cohesiveness), and moisture retention during storage. The findings demonstrate that pregelatinization optimizes starch functionality, mitigates retrogradation, and improves the overall quality of traditional corn-based foods, providing a practical approach for industrial applications.

## 1. Introduction

The increasing global demand for nutritious and sustainable food sources has increased the production of corn worldwide. Corn is a high-yield crop [1] that is rich in carbohydrates, dietary fiber, vitamins (such as B vitamins), essential minerals (such as magnesium and phosphorus), and bioactive compounds (such as phenolic acids and carotenoids), making it a valuable resource for food security [2]. China is one of the largest producers of corn globally, with an annual production of approximately 274 million metric tons in 2022, accounting for nearly 23% of the world’s total corn production. Despite this significant output, corn is predominantly used in animal feed and industrial processing, with only a limited portion (about 10%) directly consumed by humans as food [3]. This underutilization is attributable to the coarse texture and suboptimal processing characteristics of corn grits, which restrict their use in traditional Chinese foods [4]. Cooked rice wrapped in plant leaves, a culturally significant item with a long history in China, is a traditional corn-based food. Developing new types of cooked corn wrapped in plant leaves using corn grits instead of glutinous rice expands the corn food market and improves the comprehensive utilization of corn. However, corn grits have several challenges due to their hardcore and inferior texture, significantly impacting the palatability and marketability of their products [4]. Therefore, improving the quality of corn grits to enhance the sensory and textural properties of cooked waxy corn wrapped in plant leaves is urgent and crucial.

Various modification techniques, including physical, chemical, and biological methods, have been developed to enhance the functional properties of corn grits. These techniques aim to improve key properties of corn grits, such as gelatinization, swelling power, and water-holding capacity, which are crucial for food applications [5]. For instance, thermal treatments such as extrusion and steam cooking have been successfully applied to modify corn grits, resulting in improved texture and digestibility [6,7]. In addition, chemical modifications such as crosslinking and enzymatic treatments have been used to tailor the properties of corn grits to specific food applications [8]. Despite these advancements, the use of modified corn grits in traditional food products is still limited. Current modification techniques often fail to meet the specific requirements of certain traditional recipes, such as achieving full gelatinization and an appealing texture [9]. Consequently, further research is needed to optimize the modification of corn grits for a wider range of applications in traditional food systems.

The undesirable texture of cooked waxy corn wrapped in plant leaves may be attributed to the partial gelatinization of corn starch [10,11]. Gelatinization is a critical process that affects the texture and palatability of starchy foods [12]. Partial gelatinization can lead to a hardcore and poor mouthfeel, which are major drawbacks in cooked waxy corn wrapped in plant leaves [13]. These challenges can be addressed through pregelatinization treatment, which is a process that involves partial or complete gelatinization of starch before its use. Pregelatinization has been successfully applied to various cereal grains (e.g., wheat, rice, sorghum, and millet) and legumes (e.g., chickpeas, lentils, and mung beans), significantly improving their functional properties and end-product quality [14,15]. For instance, pregelatinized wheat improves the texture of bakery products because its flour exhibits enhanced swelling power and reduced retrogradation [16,17]. Similarly, pregelatinized rice can improve the quality of rice-based products by increasing the degree of gelatinization and water-holding capacity [18]. In addition, pregelatinized sorghum and millet flours exhibit improved functional properties such as increased solubility and reduced hardness in food products [19]. Therefore, pregelatinization can enhance the gelatinization degree and water-holding capacity of starches, making it a promising strategy for improving the quality of cooked waxy corn wrapped in plant leaves [20,21]. This research provides valuable insights into the modification of corn grits and contributes to the development of high-quality corn-based traditional foods.

## 2. Materials and Methods

### 2.1. Materials

The materials used in this study included waxy corn kernels (Suyunuo 5, a waxy corn variety, and Sukenuo 1801, another waxy corn variety developed in Jiangsu, China) harvested at the mature stage and provided by the Jiangsu Academy of Agricultural Sciences. The corn kernels were stored at 4 °C until use. All chemical reagents used were purchased from Sinopharm Chemical Reagent Co., Ltd. (Shanghai, China), and they were of analytical grade. Taka amylase (≥400 U/mL) was sourced from Sigma-Aldrich (St. Louis, MO, USA).

### 2.2. Pregelatinization Process

The pregelatinization process was optimized following the procedure reported in previous studies [14,15]. Corn kernels were soaked in distilled water at 55 °C for 2 h to facilitate hydration. After the kernels were soaked, they were steamed for 30 min (steam temperature maintained at 100 °C) to achieve partial gelatinization using a standard kitchen steamer (ZN28YC808-130, Supor, Hangzhou, China). Then, the steamed kernels were dried in a hot air oven (UN110, Memmert, Schwabach, Germany) at 45 °C until a constant weight was achieved. The dried kernels were ground into corn grits using a laboratory-scale grinder (JHF1000A, Kunming Iron & Steel, Kunming, China) equipped with a 40-mesh screen to ensure uniform particle size. The pregelatinized corn grits were subsequently stored in airtight containers at room temperature until further use.

### 2.3. Determination of the Pasting Properties of Corn Grits

The pasting properties of corn grits were determined using a Rapid Visco Analyzer (RVA, StarchMaster2, Perten Instruments, Hägersten, Sweden). Corn grits (3.5 g) were dispersed in 25 mL of distilled water, heated from 50 °C to 95 °C at a rate of 6 °C/min, held at 95 °C for 5 min, cooled to 50 °C at a rate of 6 °C/min, and held at 50 °C for 2 min. The gelatinization temperature (T_o_), peak viscosity, trough viscosity, final viscosity, breakdown value, and setback value were recorded.

### 2.4. Determination of Thermodynamic Properties of Corn Grits

The thermodynamic properties of corn grits were determined using differential scanning calorimetry (DSC, DISCOVERY DSC25, TA Instruments, New Castle, DE, USA). Samples (2 mg) were sealed in aluminum pans and heated from 25 °C to 120 °C at a rate of 10 °C/min under a nitrogen flow of 50 mL/min. The gelatinization onset temperature (T_o_), peak temperature (T_p_), conclusion temperature (T_c_), and gelatinization enthalpy (ΔH_g_) were recorded.

### 2.5. Determination of Solubility, Swelling Power, and Water-Holding Capacity of Corn Grits

The solubility, swelling power, and water-holding capacity of corn grits were determined following the modified method described by Li [22]. First, corn grit powder was accurately weighed in a 5 mL centrifuge tube pre-constructed to a constant weight. Then, the sample concentration was fixed at 1% (m/m, on a dry basis), and the sample was heated in a water bath at 55 °C for 1 h with shaking. Afterward, the suspension was centrifuged at 3000 rpm for 30 min, and the supernatant was transferred to a constant-weight aluminum box and heated in an oven at 105 °C to a constant weight. The mass of the dried aluminum box and the mass of the centrifuge tube containing the precipitate were weighed, and the solubility, swelling power, and water-holding capacity were calculated according to the following methods.

(1) $$\mathrm{Solubility}\;(\%)=\frac{A}{W}\;\times \;100$$

(2) $$\mathrm{Swelling}\;\mathrm{Power}=\frac{P\times 100}{W\times (100-S)}$$

(3) $$\mathrm{Water-}\mathrm{Holding}\;\mathrm{Capacity}=\frac{P-W}{W}$$

where A, P, and W represent the mass of the sample contained in the supernatant (g), the mass of the precipitate (g) and the mass of sample on a dry basis (g), respectively.

### 2.6. Determination of Corn Grit Regeneration Degree

The regeneration degree of corn grits was determined using the modified method of Temsiripong [23]. First, the thermodynamic properties of the samples from Section 2.4 were re-evaluated after DSC pasting and storing at 4 °C for 7 d. The enthalpy of regrowth (ΔHr) was obtained, and the degree of regrowth (RD) of starch was calculated using the ratio of the enthalpy of regrowth to that of the thermal enthalpy.

(4) $$\mathrm{Retrogradation}\;\mathrm{Degree}\;(\%)=\frac{\Delta \mathrm{Hr}}{\Delta \mathrm{Hg}}\times 100$$

### 2.7. Crystallinity Analysis of Corn Grits

X-ray diffraction (XRD, D8 ADVANCE, Bruker, Karlsruhe, Germany) was used to determine the crystallinity of the samples following the modified method reported by Wang [24]. First, an appropriate amount of sample was placed in the groove of the scanning disc. Then, the sample was scraped and compacted for measurement. The measurement was performed under the following conditions: diffraction angle (2θ) scanning range of 5–40°, scanning speed of 5°/min, detection step width of 0.05°, tube current of 10 mA, and tube pressure of 30 kV. Analysis was performed using MDI Jade 6 software, and the degree of crystallinity was defined as the ratio of the area of the crystalline zone to the total diffracted area.

### 2.8. Determination of Short-Range Orderliness of Corn Grits

The short-range orderliness of corn grits was determined following the modified method reported by Bie [25]. First, corn grit powder and anhydrous potassium bromide were mixed in a ratio of 60:1 (m/m), ground, and pressed in a mortar. The sample transmittance was scanned using Fourier-transform infrared spectroscopy (FTIR, IS10, Thermo Fisher Scientific, Waltham, MA, USA) in the wave number range of 4000–400 cm^−1^ with 32 scans and a resolution of 4 cm^−1^. The OMNIC Fourier self-convolution conditions were set to half peak widths of 20.5 cm^−1^ and an enhancement factor of 2.0.

### 2.9. Morphological Analysis of Corn Grits

Morphological analysis of corn grits was conducted following the method reported by Dai [26]. First, the micromorphology of the sample was examined using a scanning electron microscope (SEM, SU8100, Hitachi, Tokyo, Japan). A small amount of the sample was collected and mounted on a stage with a special conductive double-sided adhesive and then analyzed at an accelerating voltage of 3 kV and a magnification of 1500 times after it was sprayed with gold using an ion sputtering device.

### 2.10. Preparation of Cooked Waxy Corn Wrapped in Plant Leaves

Dried plant leaves without damage were washed, boiled in 3 L of water for 15 min, and soaked in hot water for 20 min and then in cold water for 12 h. Corn grits were rinsed and soaked in pure water (1:2, *w*/*w*) for 2 h, drained for 30 min, and wrapped in 100 g portions following Wufangzhai’s standard method. The wrapped samples were boiled in 3.5 L of water using an induction cooker (SUPOR, China) for 40 min at atmospheric pressure (steam temperature maintained at 100 °C). Then, the samples were pressure-cooked in 3 L of water for 40 min using an electric pressure cooker (SUPOR, China). The cooked waxy corn wrapped in plant leaves was cooled to room temperature, vacuum-sealed, and stored at 4 °C.

### 2.11. Establishment of Sensory Evaluation Panel

The sensory evaluators of the cooked corn wrapped in plant leaves were recruited and screened from among faculty members and students aged 20~40 years old at the School of Food Science and Technology, Jiangnan University, with reference to GB/T16291.1-2012 [27] for the 40 sensory evaluators recruited. After consistency and attribute perception assessment, a sensory evaluation panel of cooked corn wrapped in plant leaves, including 5 men and 5 women, was finally formed. The sensory evaluation team members were all trained for a total of 30 h on basic sensory evaluation knowledge and sensory description and analysis of cooked corn wrapped in plant leaves, and the evaluators were further trained on their competence through the two-three test and sequencing experiment [28].

### 2.12. Sensory Evaluation Methods

After boiling 1.5 L of water in a stainless-steel electric steamer, the sample of cooked corn wrapped in plant leaves was placed in the steam drawer and steamed for 15 min; after the completion of the cooking of the rice dumplings, the leaves were removed, and the evaluator evaluated the appearance of the rice dumplings. The odor was evaluated by weighing 30.00 g of cooked corn wrapped in plant leaves in an 80 mL glass bottle with lid, equilibrating the temperature in 55 °C water bath for 1 h, making the odor of the rice dumplings evenly spread in the glass bottle, opening the lid of the bottle, inhaling the odor of the rice dumplings slowly, and evaluating the odor of the rice dumplings. A small stainless-steel spoon with a capacity of 2.00 g was used to scoop the cooked corn wrapped in plant leaves, and the samples were tasted and chewed; the samples stayed in the mouth for at least 5 s, and the flavor and texture of the samples were evaluated. At the end of each sample tasting, the evaluator was required to chew 1 to 2 slices of soda crackers, and then rinse the mouth with water until the mouth was thoroughly clean, with a rest interval of 5 min between samples to eliminate the residual effects of the previous sample.

The 5-point scaling method was used for evaluation, and its geometric mean was calculated as M. M is the square root of the product of the intensity of the tamale descriptor I and the frequency F.

(5) $$M=\sqrt{F\;\times \;I}$$

### 2.13. Determination of the Weights of Sensory Evaluation Indicators

Combining the vocabulary of the quantitative descriptive table of sensory attributes of cooked corn wrapped in plant leaves, and referring to the percentage scoring criteria of glutinous rice dumplings, the team members used the method of assessing the degree of importance of each sensory attribute to assign weights to the attributes of the corn dumplings in the aspects of appearance, odor, flavor, and texture and to determine the weights of each sensory evaluation indicator.

### 2.14. Determination of Gelatinization Degree of Cooked Waxy Corn Wrapped in Plant Leaves

Samples were refrigerated at −80 °C (DW-86L388, Haier, Qingdao, China) for 24 h, freeze-dried (SCIENTE-10ND, Ningbo Xinzhi Biotechnology, Ningbo, China), and ground into powder (100 mesh). A 0.50 g sample was mixed with 25 mL of distilled water in centrifuge tubes, gelatinized in boiling water for 20 min, and cooled in an ice bath. Taka amylase (3%, 2.5 mL) was added to the mixture and incubated at 37 °C for 2 h, and the reaction was terminated with 1 mol/L HCl/NaOH. The mixture was diluted, centrifuged (RJ-LD-50G, Ruijiang Instruments, Suzhou, China), and mixed with 3,5-Dinitrosalicylic acid (DNS). Absorbance at 540 nm (T-6v spectrophotometer, Nanjing Feile Instrument, Nanjing, China) was measured. The gelatinization degree was calculated as the ratio of sample absorbance to fully gelatinized sample absorbance.

### 2.15. Determination of Moisture Distribution in Cooked Waxy Corn Wrapped in Plant Leaves

Moisture distribution in cooked waxy corn was analyzed using a low-field nuclear magnetic resonance analyzer (LF-NMR, MesoMR23-060V-I, Niumag Electronics, Shanghai, China). Samples were cut into uniform 3.00 g blocks, sealed in sample bottles, and placed in a 25 mm coil. The Carr–Purcell–Meiboom–Gill sequence was applied using the following parameters: relaxation delay time (TW) of 1500 ms, number of scans of 2, echo time (TE) of 0.3 ms, and number of echoes of 15,000. Inversion parameters included a filter level of 3 and 1,000,000 iterations.

### 2.16. Pregelatinization Procedure of Cooked Waxy Corn Wrapped in Plant Leaves During Storage

Samples were prepared as described in Section 2.10 and stored at 4 °C for 0, 1, 3, 5, 7, and 9 days. At each time point, samples were analyzed for texture, moisture content, and gelatinization degree to assess quality changes. Texture analysis was performed using a texture analyzer to measure hardness, chewiness, adhesiveness, and cohesiveness. Moisture content was determined by oven-drying, while the gelatinization degree was analyzed using the DNS method as outlined in Section 2.14.

### 2.17. Data Statistics and Analysis

In this experiment, each index was measured in triplicate, taking the average value, and the data were recorded as the mean ± standard deviation. Data statistics and analysis of variance were performed using Excel 2022 and SPSS Statistics V26, and results were plotted using Origin 2024b graphing software.

## 3. Results and Discussion

### 3.1. Effect of Pregelatinization Treatment on Physicochemical Properties of Corn Grits

#### 3.1.1. Effect of Pregelatinization Treatment on the Pasting Properties of Corn Grits

**Figure 1:**
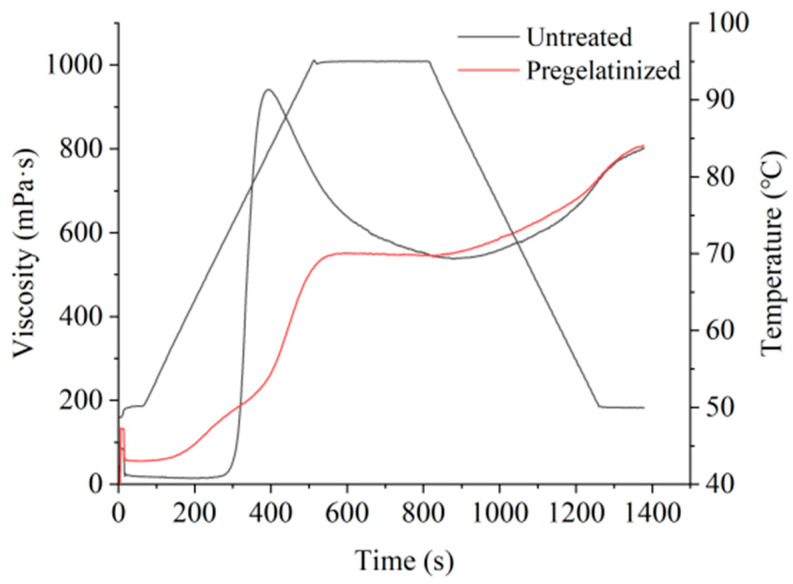
Gelatinization characteristic curves of corn grits.

The pasting characteristics and thermodynamic properties of corn grits were analyzed using RVA and DSC before and after the pregelatinization treatment was conducted (Table 1 and Table 2, Figure 1). The results showed that the pregelatinization treatment significantly reduced the paste-forming temperature, peak viscosity, disintegration value, and regrowth value of the grits. This phenomenon can be attributed to the disruption of intermolecular forces and the original granular structure of starch during pregelatinization. The treatment weakens hydrogen bonds between starch molecules, leading to partial destruction of the crystalline regions and increased molecular mobility, thereby lowering the energy required for gelatinization. These findings are consistent with studies on corn starch gelatinization, which reported similar effects of pregelatinization on viscosity properties due to structural modifications [29]. Additionally, the reduced retrogradation value indicates suppressed starch recrystallization, suggesting that pregelatinization alters the amylose–amylopectin interactions, as observed in previous research [30].

In terms of thermodynamic properties, the pregelatinization treatment reduced the onset paste-forming temperature, peak temperature, and enthalpy of pasting of the corn grits. This reduction indicated that the pregelatinized corn grits absorbed less heat at the onset of pasting and were more susceptible to pasting than untreated corn grits. The decrease in peak temperature was mainly attributed to the crystal strength and double-helix structure. The pregelatinization treatment disrupted the crystalline structure of the starch molecules, transforming them from an ordered to a disordered state and leading to the solubilization of the short-branched starch molecules [31]. The decrease in the thermal enthalpy of pasting was attributed to disruption of the double-helix structure in the crystalline region of the starch, which reduced the energy required for the phase transition [19].

#### 3.1.2. Effect of Pregelatinization Treatment on the Application Properties of Corn Grits

The solubility, swelling power, and water-holding capacity of corn grits before and after the pregelatinization treatment were determined at 95 °C. As shown in Table 3, the solubility, swelling power, and water-holding capacity of corn grits before and after the pregelatinization treatment had significant changes. The solubility of corn grits was correlated with the size of their adhesive force [32]. After pregelatinization treatment, starch granules were damaged and degraded to produce small molecules, which increased their solubility. After pregelatinization treatment, corn grits undergo pasteurization to increase the content of branched starch after the breakage of starch granules, which leads to an increase in their swelling power [33]. After pregelatinization, the starch granules lose their original integrity, and the intramolecular and intermolecular hydrogen bonding and crystal structure are destroyed, resulting in the exposure of more hydrophilic groups and the formation of hydrogen bonding with water molecules, which leads to a significant increase in the water-holding capacity [34]. The increase in swelling power and water-holding capacity of the pregelatinized treated corn grits also indicated that cooking corn wrapped in plant leaves would result in moister and better tasting corn grits.

The measured samples were stored at 4 °C for 7 d by DSC and then measured again, and the degree of regrowth was calculated. As shown in Table 4, the differences in the starting paste-forming temperature, peak temperature, and termination paste-forming temperature before and after pregelatinization of the regrowth of the corn grits were small, while the differences in the enthalpy of pasting and regrowth were large, with the enthalpy of pasting of the pregelatinized grits being 1.03 J/g, and the degree of regrowth being 45.26%, which was significantly lower than that of the untreated corn grits. Compared with the pregelatinized corn grits, the untreated corn grits required more energy to destroy the inter- and intramolecular chemical bonds in the crystalline zone after regrowth, while the pregelatinized corn grits were more stable during the storage period, with a smaller degree of regrowth and a better ability to resist regrowth.

#### 3.1.3. Effect of Pregelatinization Treatment on the Microstructure of Corn Grits

**Figure 2:**
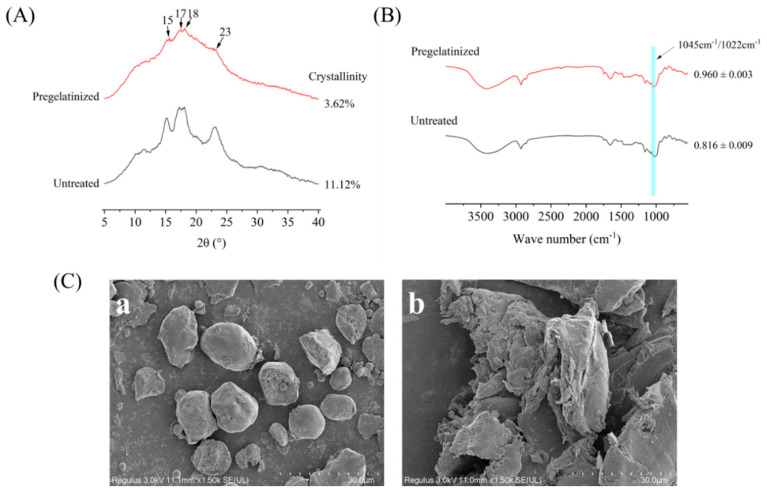
Microstructure of corn grits before and after treatment. (**A**) X-ray diffraction patterns of corn grits; (**B**) FTIR spectra of corn grits; (**C**) SEM images of corn grits ((**a**), untreated; (**b**), pregelatinized).

The effect of pregelatinization on the microstructure of corn granules could be elucidated systematically using multiscale analytical techniques. X-ray diffraction (XRD) results showed that both unpasteurized and pasteurized corn starch exhibited the characteristic A-type crystal peaks (at 15°, 17°, 18°, and 23°), but pregelatinization significantly reduced the peak intensity (Figure 2A). This suggests that, while the crystal type remains unchanged, pregelatinization disrupts the long-range ordering (i.e., crystallinity) of the starch granules. This reduction in crystallinity arises from dissociation of the starch’s double-helix branching structure, reducing the energy required for starch pasting and optimizing subsequent processing properties [35]. Fourier-transform infrared spectroscopy (FTIR) analysis showed no significant change in the starch’s peak shape before and after pregelatinization, indicating that no new chemical groups were generated. However, the decrease in the 1045 cm^−1^/1022 cm^−1^ ratio (Figure 2B) revealed weakening of the short-range ordering. This suggests breaking of hydrogen bonds and an increase in free hydroxyl groups, which leads to an increase in the proportion of amorphous structures [36]. This result is consistent with the XRD data, which together suggest that pregelatinization reduces the stability of starch by disrupting the intermolecular ordering arrangement. Scanning electron microscopy (SEM) observations directly confirmed these conclusions: unpasteurized starch granules exhibited intact polygonal or elliptical structures, whereas pre-pasteurized granules were significantly swollen and ruptured, forming amorphous flakes (Figure 2C). This morphological change is closely related to the melting of microcrystalline bundles and increased molecular disorder [37]. When the results of the three techniques are combined, it becomes apparent that pregelatinization significantly alters the microstructural properties of corn granules. This occurs by simultaneously weakening the long- and short-range ordering of the starch and inducing a transition from ordered crystals to an amorphous phase.

### 3.2. Effect of Pregelatinization Treatment on the Quality of Cooked Waxy Corn Wrapped in Plant Leaves

#### 3.2.1. Effect of Pregelatinization Treatment on the Eating Quality of Cooked Waxy Corn Wrapped in Plant Leaves

**Figure 3:**
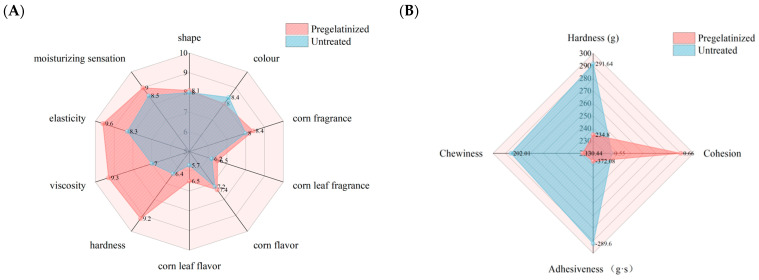
Eating quality of cooked waxy corn wrapped in plant leaves before and after treatment. (**A**) Sensory quality of cooked waxy corn wrapped in plant leaves; (**B**) texture analysis of cooked waxy corn wrapped in plant leaves.

The organoleptic quality and texture analysis revealed that pregelatinization treatment significantly enhanced the eating quality of the corn wrapped in plant leaves. Significant differences were observed in the sensory quality of the cooked waxy corn wrapped in plant leaves before and after pregelatinization treatment, particularly in hardness, viscosity, and elasticity (Figure 3A). Pregelatinization treatment reduced the hardness, increased the viscosity, and enhanced the elasticity of the cooked waxy corn wrapped in plant leaves. This phenomenon was attributed to the disruption of the branched starch double-helix structure in the crystalline region of corn grits starch caused by the treatment, leading to a reduction in the degree of starch ordering and an improvement in the degree of pasting. Compared with untreated corn grits, pregelatinized corn grits exhibited enhanced pasting properties and reduced cooking time. This improvement indicated that during the secondary cooking process of the cooked waxy corn wrapped in plant leaves, pregelatinized corn grits demonstrated a higher degree of pasting and a more complete pasting process. Consequently, this improvement in pasting quality enhanced the palatability of the corn wrapped in plant leaves. The textural indexes of the cooked waxy corn wrapped in plant leaves (Figure 3B) revealed significant differences in hardness, chewiness, adhesion, and cohesion of the corn wrapped in plant leaves before and after the pregelatinization treatment. Of particular note was the significant decrease in hardness (19.49%) and chewiness (35.43%) of the cooked waxy corn wrapped in plant leaves after the pregelatinization treatment, while adhesion (28.48%) and cohesion (20%) increased. This finding showed that the pregelatinization treatment increased the degree of starch pasting and that the pasting process was more complete during the secondary cooking process. As a result, starch dissolution and rupture in the cooked waxy corn wrapped in plant leaves increased, thereby making them softer and more glutinous and improving the adhesion and mouthfeel properties. The pregelatinization treatment enhanced the degree of gelatinization of the cooked waxy corn wrapped in plant leaves and significantly optimized their textural qualities, resulting in a better taste, which was consistent with the results of the organoleptic quality analysis.

#### 3.2.2. Effect of Pregelatinization Treatment on the Pasting Characteristics of Cooked Waxy Corn Wrapped in Plant Leaves

**Figure 4:**
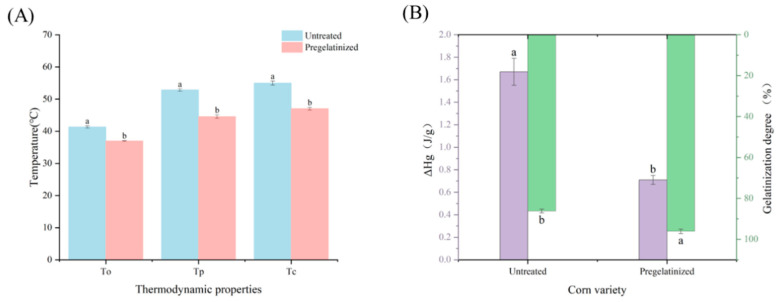
Pasting characteristics of cooked waxy corn wrapped in plant leaves before and after treatment. (**A**) Thermodynamic properties of cooked waxy corn wrapped in plant leaves. (**B**) Gelatinization degree and ΔHg of cooked waxy corn wrapped in plant leaves.

The pasting characteristics of corn wrapped in plant leaves before and after a pregelatinization treatment were analyzed. The results showed that the pregelatinization treatment significantly enhanced the pasting degree of corn wrapped in plant leaves. As shown in Figure 4B, the degree of pasting of the pregelatinized corn wrapped in plant leaves attained 96.11% under the same cooking conditions, which was considerably higher than that of the untreated corn wrapped in plant leaves. This finding revealed that the pregelatinization treatment compromised part of the double-helix structure in the crystalline region of the starch, thereby reducing the energy required for phase transition. Consequently, the cooked waxy corn wrapped in plant leaves was rapidly and fully gelatinized during the secondary cooking and gelatinization process. A previous study has shown that the degree of pasting significantly correlates with the sensory textural quality and the regrowth resistance of the cooked waxy corn wrapped in plant leaves. This correlation further substantiates the hypothesis that the pregelatinization treatment enhances the overall quality of corn wrapped in plant leaves and regrowth resistance. Furthermore, the pregelatinization treatment significantly impacted the thermodynamic characteristics of the cooked waxy corn wrapped in plant leaves (Figure 4A). In addition, the pregelatinization treatment reduced the onset, paste-forming, peak, and termination temperatures of the cooked waxy corn wrapped in plant leaves. This reduction was attributed to a change in the binding force between molecular chains in the crystalline and amorphous regions caused by the increased pasting of the corn grits, thereby decreasing the percentage of the crystalline region [38]. The enthalpy of the pasting of corn wrapped in plant leaves prepared from pregelatinization processed corn grits was even lower, at approximately 0.71 J/g. This finding showed that pre-pasteurization and cooking effectively destroyed the starch crystalline region of the branched-chain starch double-helix structure, increased the degree of starch disorder, and reduced the thermal energy required for the third heating phase, suggesting that minimal heat is sufficient to complete pasting. The decrease in the initial paste-forming temperature indicated that the samples of corn wrapped in plant leaves prepared with pregelatinized corn grits were more easily processed into a paste, with starch gelatinizing at a lower temperature.

#### 3.2.3. Effect of Pregelatinization Treatment on Moisture Content and Moisture Distribution of Cooked Waxy Corn Wrapped in Plant Leaves

**Figure 5:**
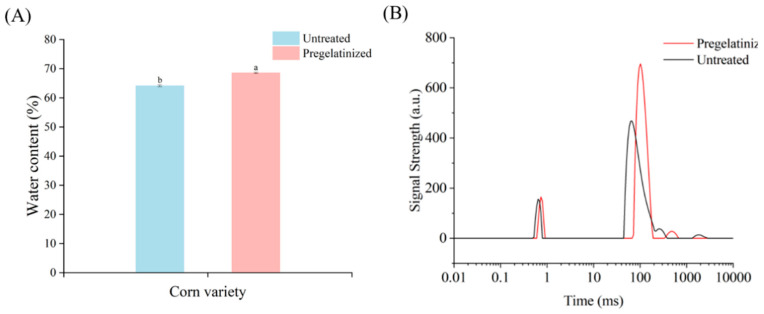
Moisture content and moisture distribution of cooked waxy corn wrapped in plant leaves before and after treatment. (**A**) Moisture content of cooked waxy corn wrapped in plant leaves; (**B**) moisture status of cooked waxy corn wrapped in plant leaves.

The moisture content of the cooked waxy corn wrapped in plant leaves prepared from corn grits before and after pregelatinization treatment was determined. The results showed that the pregelatinization treatment significantly affected the moisture content of the cooked waxy corn wrapped in plant leaves (Figure 5A). The moisture content of the cooked waxy corn wrapped in plant leaves prepared from pregelatinized corn grits was 6.88% higher than that of the untreated corn wrapped in plant leaves. This discrepancy can be mainly attributed to the extent of starch pasting and its capacity for water retention. The pregelatinization treatment enhanced the pasting degree and water-holding capacity of the cooked waxy corn wrapped in plant leaves, thereby increasing the starch–water binding capacity. This enhancement positively affected the taste and quality of the cooked waxy corn wrapped in plant leaves. The cooked waxy corn wrapped in plant leaves exhibited a softer texture and enhanced elasticity, and the overall sensory quality and textural properties were significantly improved.

Regarding moisture distribution, the cooked waxy corn wrapped in plant leaves before and after pregelatinization treatment exhibited divergent results (Figure 5B). The moisture-binding states of the cooked waxy corn wrapped in plant leaves can be classified into three types, T_21_, T_22_, and T_23_, and three different fractions, M_21_, M_22_, and M_23_. As shown in Table 5, the transverse relaxation times of T_21_, T_22_, and T_23_ were higher in the cooked waxy corn wrapped in plant leaves after pregelatinization treatment, indicating that the strongly bound water migrated to the weakly bound and free water in the corn wrapped in plant leaves. This phenomenon indicates that the binding of starch, protein, and other components to water was reduced, and the mobility of water increased, which further reduced the binding force of water. Meanwhile, the peak areas of M_21_ and M_22_ increased, indicating that the pregelatinization treatment destroyed the microstructure of the corn grits and the microstructure inside the cooked waxy corn wrapped in plant leaves during the secondary gelatinization process. Thus, the water-binding force decreased, the water mobility increased, and the corn wrapped in plant leaves was fully gelatinized.

### 3.3. Effect of Pregelatinization Treatment on the Quality of Cooked Waxy Corn Wrapped in Plant Leaves During Storage

#### 3.3.1. Effect of Pregelatinization on the Textural Characteristics of Cooked Waxy Corn Wrapped in Plant Leaves During Storage

**Figure 6:**
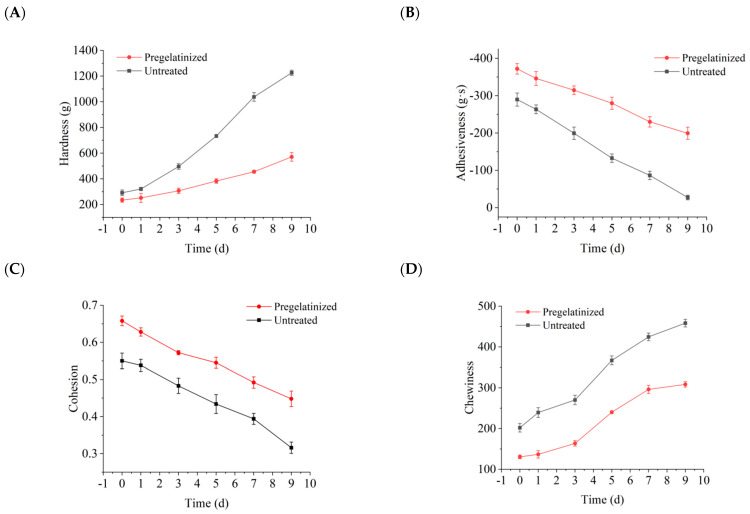
Texture analysis changes in cooked waxy corn wrapped in plant leaves before and after pretreatment. (**A**) Hardness; (**B**) adhesiveness; (**C**) cohesion; (**D**) chewiness.

The experiment involved the preparation of corn grits, which were cooked and wrapped in plant leaves. The corn grits used in the experiment were either pregelatinized or untreated, and the storage conditions were set at 4 °C for 0, 1, 3, 5, 7, and 9 d. Then, the qualitative and structural properties of the corn grits were determined. The analysis results are presented in Figure 6. During the same storage time, the quality of corn wrapped in plant leaves prepared from pregelatinized corn grits slightly changed compared with that of corn wrapped in plant leaves prepared from untreated grits, with a slower tendency of quality decline. The hardness of pregelatinized cooked waxy corn wrapped in plant leaves increased by 143.07%, while the hardness of the untreated corn wrapped in plant leaves increased by 320.27%. The pregelatinization treatment improved the textural characteristics of the corn wrapped in plant leaves by increasing the degree of pasting, resulting in a more gradual increase in hardness. This finding revealed that pregelatinization improved the anti-aging properties and palatability of cooked waxy corn wrapped in plant leaves during storage, thereby effectively enhancing its quality. This result is consistent with the results of the pre-sensory evaluation.

#### 3.3.2. Effect of Pregelatinization Treatment on the Moisture Content of Cooked Waxy Corn Wrapped in Plant Leaves During Storage

**Figure 7:**
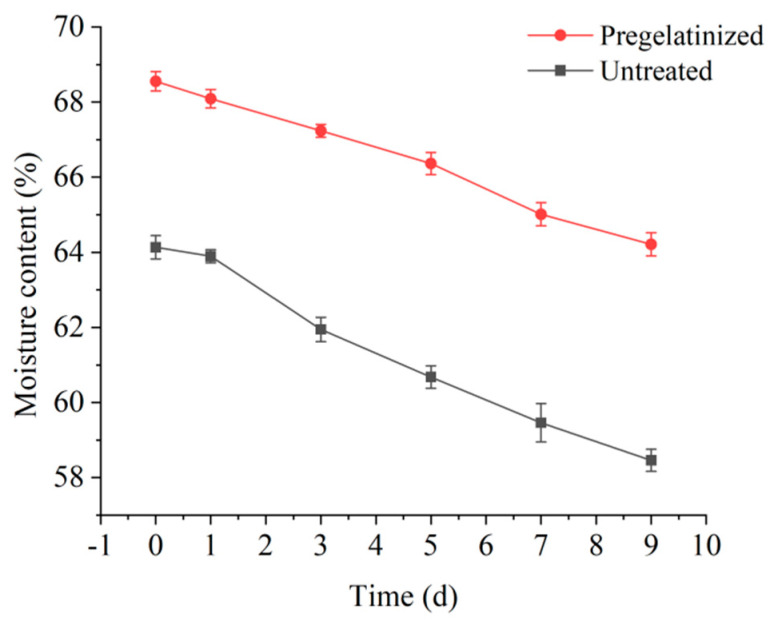
Moisture content changes in cooked waxy corn wrapped in plant leaves before and after pretreatment.

The experiment involved the preparation of corn wrapped in plant leaves from pregelatinized and untreated corn grits. The corn was stored at 4 °C for 0, 1, 3, 5, 7, and 9 d. Then, the moisture content of the corn was analyzed. The analysis results are presented in Figure 7.

The moisture content of the pregelatinized and unpregelatinized cooked waxy corn wrapped in plant leaves prepared from corn grits decreased with increasing storage time. The pregelatinized corn wrapped in plant leaves exhibited a slower rate of moisture loss than the unpregelatinized corn wrapped in plant leaves, with a reduction of 6.33% over the storage period. However, the moisture content in the untreated corn wrapped in plant leaves significantly decreased to 8.84%, indicating that the pregelatinized corn wrapped in plant leaves underwent a less effective process of water evaporation during storage. After pregelatinization, the degree of pasting of the corn wrapped in plant leaves was significantly higher and was almost close to complete pasting. This phenomenon indicated stronger starch–water interaction and improved water-retention capacity in the corn wrapped in plant leaves, resulting in a lower degree of regrowth of the cooked waxy corn wrapped in plant leaves after pregelatinization of the grits. In addition, this process resulted in a softer and moister texture with better organoleptic quality, indicating that pregelatinization effectively improved the quality of the corn wrapped in plant leaves.

## 4. Conclusions

The present study investigated the impact of pregelatinization treatment on both the physicochemical characteristics of corn grits and the quality of cooked waxy corn wrapped in plant leaves. The results demonstrated that pregelatinization significantly enhanced the physicochemical properties of corn grits while improving the quality of cooked waxy corn. This treatment optimized starch functionality by reducing retrogradation, increasing solubility, and improving water-holding capacity, collectively enhancing texture and moisture retention in the final product. These findings highlight pregelatinization as an effective strategy for addressing challenges in traditional corn-based food production. The study establishes a theoretical foundation for understanding modified starch behavior and its practical applications in food processing. Future research should explore the method’s scalability for industrial applications and its adaptability to other starch-based foods, thereby advancing corn utilization in human nutrition and food innovation.

## Figures and Tables

**Table 1:** Pasting property parameters of corn grits before and after treatment.

Corn Grits Sample	Gelatinization Temp (°C)	Peak Viscosity (mPa·s)	Trough Viscosity (mPa·s)	Final Viscosity (mPa·s)	Breakdown (mPa·s)	Setback (mPa·s)
Untreated	72.90 ± 0.53 ^a^	941.60 ± 10.08 ^a^	538.42 ± 6.13 ^a^	801.40 ± 7.38 ^a^	403.83 ± 17.85 ^d^	274.83 ± 5.22 ^a^
Pregelatinized	66.30 ± 0.24 ^b^	552.71 ± 11.81 ^a^	544.70 ± 8.54 ^a^	807.60 ± 8.47 ^a^	8.20 ± 2.47 ^b^	154.52 ± 3.04 ^b^

Note: Values are expressed as means ± standard deviations. In the same column, data that do not share lowercase letters are significantly different, *p* < 0.05.

**Table 2:** Thermodynamic properties of corn grits before and after treatment.

Corn Grits Sample	T_o_ (°C)	T_p_ (°C)	T_c_ (°C)	ΔHg (J/g)
Untreated	69.29 ± 0.47 ^a^	77.86 ± 1.33 ^a^	85.72 ± 3.74 ^a^	9.66 ± 0.14 ^a^
Pregelatinized	45.35 ± 0.33 ^b^	55.74 ± 0.30 ^b^	56.30 ± 0.18 ^b^	2.27 ± 0.09 ^b^

Note: Values are expressed as means ± standard deviations. In the same column, data that do not share lowercase letters are significantly different, *p* < 0.05.

**Table 3:** The solubility, swelling power, and water-holding capacity of corn grits before and after treatment.

Corn Grits Sample	95 °C		
	Solubility (%)	Swelling Power (g/g)	Water-Holding Capacity (g/g)
Untreated	20.10 ± 0.71 ^b^	21.91 ± 0.76 ^b^	19.17 ± 0.45 ^b^
Pregelatinized	28.80 ± 0.28 ^a^	31.15 ± 0.50 ^a^	23.18 ± 1.15 ^a^

Note: Values are expressed as means ± standard deviations. In the same column, data that do not share lowercase letters are significantly different, *p* < 0.05.

**Table 4:** Retrogradation of corn grits before and after treatment.

Corn Grits Sample	T_o_ (°C)	T_p_ (°C)	T_c_ (°C)	ΔHr (J/g)	RD (%)
Untreated	41.33 ± 0.43 ^b^	55.70 ± 0.72 ^a^	65.56 ± 1.55 ^a^	5.42 ± 0.16 ^a^	56.06
Pregelatinized	43.58 ± 0.63 ^a^	56.33 ± 0.19 ^a^	63.76 ± 0.34 ^a^	1.03 ± 0.07 ^b^	45.26

Note: Values are expressed as means ± standard deviations. In the same column, data that do not share lowercase letters are significantly different, *p* < 0.05.

**Table 5:** Moisture distribution of cooked waxy corn wrapped in plant leaves before and after pretreatment.

Corn Grits Sample	T_21_ (ms)	M_21_ (%)	T_22_ (ms)	M_22_ (%)	T_23_ (ms)	M_23_ (%)
Untreated	0.474 ± 0.030 ^b^	7.10 ± 0.62 ^b^	46.342 ± 0.405 ^b^	82.34 ± 0.51 ^b^	225.624 ± 6.485 ^b^	4.87 ± 0.04 ^b^
Pregelatinized	0.602 ± 0.012 ^a^	9.45 ± 0.50 ^a^	68.020 ± 0.413 ^b^	91.94 ± 0.57 ^a^	315.325 ± 8.413 ^b^	1.69 ± 0.02 ^a^

Note: Values are expressed as means ± standard deviations. In the same column, data that do not share lowercase letters are significantly different, *p* < 0.05.

## Data Availability

The original contributions presented in this study are included in the article. Further inquiries can be directed to the corresponding author.
